# Neurons can upregulate Cav-1 to increase intake of endothelial cells-derived extracellular vesicles that attenuate apoptosis via miR-1290

**DOI:** 10.1038/s41419-019-2100-5

**Published:** 2019-11-18

**Authors:** Kang-Yi Yue, Pei-Ran Zhang, Min-Hua Zheng, Xiu-Li Cao, Yuan Cao, Yi-Zhe Zhang, Yu-Fei Zhang, Hai-Ning Wu, Zhi-Hong Lu, Liang Liang, Xiao-Fan Jiang, Hua Han

**Affiliations:** 1 State Key Laboratory of Cancer Biology, Fourth Military Medical University, 710032 Xi’an, China; 2 Department of Biochemistry and Molecular Biology, Fourth Military Medical University, 710032 Xi’an, China; 3 Department of Neurosurgery, Xijing Hospital, Fourth Military Medical University, 710032 Xi’an, China; 4 Department of Medical Genetics and Developmental Biology, Fourth Military Medical University, 710032 Xi’an, China; 5 Department of Anesthesiology and Perioperative Medicine, Xijing Hospital, Fourth Military Medical University, 710032 Xi’an, China

**Keywords:** Apoptosis, Stroke

## Abstract

Extracellular vesicles (EVs) including exosomes can serve as mediators of cell–cell communication under physiological and pathological conditions. However, cargo molecules carried by EVs to exert their functions, as well as mechanisms for their regulated release and intake, have been poorly understood. In this study, we examined the effects of endothelial cells-derived EVs on neurons suffering from oxygen-glucose deprivation (OGD), which mimics neuronal ischemia-reperfusion injury in human diseases. In a human umbilical endothelial cell (HUVEC)–neuron coculture assay, we found that HUVECs reduced apoptosis of neurons under OGD, and this effect was compromised by GW4869, a blocker of exosome release. Purified EVs could be internalized by neurons and alleviate neuronal apoptosis under OGD. A miRNA, miR-1290, was highly enriched in HUVECs-derived EVs and was responsible for EV-mediated neuronal protection under OGD. Interestingly, we found that OGD enhanced intake of EVs by neurons cultured in vitro. We examined the expression of several potential receptors for EV intake and found that caveolin-1 (Cav-1) was upregulated in OGD-treated neurons and mice suffering from middle cerebral artery occlusion (MCAO). Knock-down of Cav-1 in neurons reduced EV intake, and canceled EV-mediated neuronal protection under OGD. HUVEC-derived EVs alleviated MCAO-induced neuronal apoptosis in vivo. These findings suggested that ischemia likely upregulates Cav-1 expression in neurons to increase EV intake, which protects neurons by attenuating apoptosis via miR-1290.

## Introduction

Stroke is one of the major health threats worldwide with high mortality and morbidity, leading to ~5 million deaths per year^1^. Rapid deprivation of oxygen and nutrients immediately after ischemia, as well as the following reperfusion damage in many cases, lead to massive necrotic and apoptotic neuronal death attributed to reduction of ATP, release of excitatory neurotransmitters, burst of reactive oxygen species (ROS), intracellular calcium overload, and local inflammation. As a consequence, these pathological insults also frequently lead to persistent disability in patients surviving a stroke^2^. Therefore, neuronal protection and regeneration therapies have been urgently required, however, an efficient way to protect neurons from cell death during and after stroke has not been satisfying so far^3–6^.

Extracellular vesicles (EVs), including microvesicles, ectosomes, exosomes, apoptotic bodies, and so on, are highly heterogeneous membranous vesicles with lipid bilayer and various cargos, and are suggested as novel mediators of intercellular communication^7^. EVs are classified according to different standards, for instance, exosomes or small EVs (sEVs) represent those with the size between 30 and 150 nm. Exosomes originate from endosomes through a ceramide- and/or endosomal sorting complex required for transport (ESCRT)-dependent pathway, and are secreted into various body fluids by almost all types of cells via the way of exocytosis after fusion of multivesicular bodies (MVBs)^8,9^. EVs exert their functions by transmitting their cargos including proteins, nucleic acids, and lipid molecules, into recipient cells^10–12^. Thus, after secretion into various body fluids including blood, cerebrospinal fluid, urine, saliva, and breast milk, EVs are internalized by recipient cells by mechanisms similar to receptor–ligand interaction^13^. A panel of molecules including Flotillin-1 (Flot-1), caveolin (Cav)-1, Pak-1, Rac-1, and Dynamin-2 (Dyn-2), has been identified as receptors mediating EV/exosomal intake by various types of cells^14^. After internalization through endocytosis, phagocytosis, and/or direct fusion, EVs/exosomes are able to deliver their cargos into cytosol, and thereby modify the physiological states of recipient cells^8^.

As EVs can be prepared in large scale by a variety of methods, recent studies have been focused on the therapeutical use of EVs in different human diseases including stroke, considering their advantages in delivering bio-active molecules into brain^15–17^. For instance, functionalized EVs derived from mesenchymal stem cells (MSCs) can decrease the volume of the ischemic region in the brain after ischemic stroke^18^. Moreover, EVs/exosomes have also been shown to regulate cerebral angiogenesis, neurogenesis, neuronal plasticity, and glia in the central nervous system (CNS) under various conditions^19–23^. Endothelial cells (ECs) consist one of the largest cell population in CNS and locate in close proximity to neurons and neural stem cells (NSCs)^24,25^. ECs not only form blood vessels in brain to provide nutrients to and bring metabolites away from neural cells, but also secrete bioactive molecules to regulate different cell populations in CNS by angiocrine^26,27^. In stroke, ECs and the relevant angiocrine functions were shown to participate in the onset, progression, and post-injury neural regeneration of the disease^28–30^. In recent years, roles of EVs especially exosomes secreted by ECs have been recognized as important mechanisms mediating EC–neural cell interactions^31,32^. We have shown that ECs-derived exosomes could promote self-renewal, proliferation, and suppress apoptosis of NSCs in mice^31^. However, a role and the underlying mechanisms of ECs-derived EVs in neuronal damage upon ischemia and reperfusion injury (IRI) have not been fully explored. In this study, we accessed this question using human umbilical vein endothelial cells (HUVECs)-derived EVs. We show that EC-derived EVs could protect neurons after oxygen and glucose deprivation (OGD) insult in vitro and brain IRI in vivo, and this effect was likely mediated by miR-1290 carried by EVs. Interestingly, we found that OGD treatment upregulated the expression of Cav-1, a receptor mediating exosomal intake, in neurons. These findings suggested that neurons under IRI could increase their survival by actively upregulating Cav-1 to enhance intake of EVs/exosomes derived from ECs.

## Materials and methods

### Human tissues and mice

Human umbilical cord biopsies were obtained from the Department of Gynecology and Obstetrics, Xijing Hospital. Informed consent was obtained from individuals donating their samples. The protocols involving human samples were approved by the Ethics Committee of Xijing Hospital.

C57BL/6 male mice were maintained under specific pathogen-free conditions. MCAO was performed with mice of 8-weeks-old following a standard protocol^33^. The occlusion was maintained for 60 min before starting reperfusion, and mice were maintained for 24 h before further analysis. For triphenyltetrazolium chloride (TTC) staining, brain slices were immersed in 2% TTC solution (Solarbio, Beijing, China) for 30 min at 37 °C in dark. Pale areas were quantitatively measured. In some experiments, randomly selected mice injured by MCAO were injected intracranially with 1 μL of EV preparations (5 μg/μL) or PBS at the site near CA1–CA2 of hippocampus (AP: 2.0 mm, ML: 1.7 mm, DV: 1.35 mm) of the impaired hemisphere under the navigation of a murine brain stereotaxic apparatus (RWD68000, RWD Life Sciences Co., Ltd, Shenzhen, China). Both EVs and PBS were pre-labeled (see below) to track the injection sites. Subsequently, mice were maintained routinely for 24 h, and brain sections were prepared for TUNEL assay. To quantify apoptosis, the injection site in each hemisphere was marked, and samples with an acceptable injection site nearing CA1–CA2 of hippocampus were analyzed further. With the injection site as the center, a circle was made to include the CA1–CA2 of hippocampus near the injection site (Supplementary Fig. S1). TUNEL^+^ cells of CA1–CA2 within the circle were counted and compared blindly. All experiments were approved and followed the guidelines issued by the Animal Experiment Administration Committee of the Fourth Military Medical University.

### Cell culture

Primary HUVECs were obtained from umbilical vein as described^34^. Single cell suspensions were cultured in endothelial cell medium (ECM, Sciencell, San Diego, CA) supplemented with 5% fetal bovine serum (FBS), 1% endothelial cell growth supplements (ECGS), 100 U/mL penicillin, and 100 μg/mL streptomycin in a humidified atmosphere with 5% CO_2_ at 37 °C. Cells between passages 3 and 5 were used in experiments.

Primary hippocampal neurons were isolated from mouse embryos at E17.5. In brief, the hippocampus was dissected and cut into pieces and minced gently. Tissues were digested in 0.25% EDTA-free trypsin for 20 min at 37 °C with intermittent gentle shaking every 5 min. After removing tissue debris, cells were resuspended in Dulbeccoo’s modified Eagle’s medium (DMEM) supplemented with 20% FBS and seeded in culture dishes pre-coated with poly-l-lysine (PLL) (50 μg/mL, Sigma-Aldrich, PA) at 37 °C for 4 h to get adherence. Subsequently, neurons were washed with PBS to remove FBS and cultured in neurobasal medium containing 1% Glutamax and 2% B27 (Gibco, CA) at 37 °C in a humidified 5% CO_2_ incubator. The medium was half changed at day 3 and maintained for 7 days. For OGD, the medium was removed. Cells were rinsed with PBS three times and cultured with glucose-free DMEM pre-gassed with 94% N_2_, 1% O_2_, and 5% CO_2_ in a low oxygen incubator (94% N_2_, 1% O_2_, and 5% CO_2_) at 37 °C for 1.5 h. Cells were then cultured in complete medium again at 37 °C in a regular incubator for 24 h.

For co-culture, HUVECs were seeded in the upper chamber of a 24-well transwell system (pore size: 0.4 μm, Corning #353095, NY) and cultured up to 80% confluence. HUVECs were rinsed with PBS for three times and loaded to neurons cultured in a 24-well plate with the medium of Neurobasal, 2% B27, and 1% glutamine. In some cases, neurons were pretreated by OGD. GW4869, a blocker of exosomal secretion, was used at the concentration of 10 μM^35^.

### EV isolation and labeling

EVs were isolated from HUVECs using a kit following the manufacturer’s protocol (Sigma-Aldrich, Cat #, BCBT8582)^31,36^. Briefly, HUVECs were cultured to 80% confluence, and the culture medium was changed into serum-free ECM with 1% ECGS and cultured further for 72 h. The culture medium was collected and successively centrifuged at 500×*g* for 5 min and 3000×*g* for 30 min to remove cell debris. The supernatants were then filtrated through a 0.22-μm filter (Millipore, CA), and mixed with the PEG6000 working solution to the final concentration of 12% PEG6000. The mixtures were incubated at 4 °C for 12 h followed by centrifuging at 12,000×*g* for 1 h. The supernatants were discarded completely, and pellets were resuspended in PBS and washed with PBS twice. EVs was quantified using the microtiter plate BCA assay (Thermo Scientific Scientific, Waltham, MA) according to the provided protocol. The size of EVs was determined using ZETASIZER Nano series-Nano-ZS (Malvern, England, UK) and transmission electron microscopy (TEM)^31^. For labeling, EVs in PBS were mixed with DiI (Molecular Probes, MA) at a concentration of 20 μM and incubated at room temperature for 20 min. The mixtures were then centrifuged at 12,000×*g* for 30 min and washed with PBS for three times. For PKH-67 labeling, PKH-67 (BestBio, Shanghai, China) were diluted 10 times by diluent and then diluted 25 times by EV samples in PBS. After incubated at 4 °C for 15 min, the mixture was used for the injection.

For incubation with cultured neurons, purified EVs were resuspended in complete neuronal medium to the final concentration of 10 μg/μL. The mixtures were loaded to cultured neurons and incubated for 12 h. Cells were rinsed with PBS, and cultured routinely in neuronal medium further.

### Immunofluorescence staining

Cells were fixed by 4% paraformaldehyde (PFA) and rinsed with PBS for three times, followed by permeabilization with 0.2% Triton X-100 for 10 min. Samples were blocked by 1% BSA for 30 min and incubated with primary antibodies overnight at 4 °C. Cells were then incubated with Dylight 488-conjugated or Dylight 594-conjugated secondary antibodies (Genetex, Alton, CA) or for 1 h. Washing with PBS was performed between each staining steps. Nuclei were counter-stained with Hoechst for 5 min. Cells were observed under a fluorescence microscope (BX51, Olympus, Tokyo, Japan). For imaging, the transparent observation chamber was sterilized, pre-gassed with humidified mixed gas (5% CO_2_), and pre-heated to 37 °C. Neurons were incubated with DiI-labeled EVs for 30 min, and distributed in the observation chamber. Cell images were recorded under a fluorescence microscope (Nikon, Tokyo, Japan) for 12 h.

### Cell death assays

Apoptosis was detected using terminal deoxynucleotidyltransferase-mediated dUTP nick end labeling (TUNEL) with the DeanEnd^TM^ Fluorometric TUNEL System kit (Promega, WI) following the manufacturer’s instructions. In brief, neurons were seeded on PLL-coated glass coverslips. Cells were rinsed with PBS for three times, fixed with 4% PFA for 15 min and permeabilized with 0.1% Triton X-100 and 0.1% sodium citrate solution. Cells were then equilibrated for 10 min at room temperature in the equilibration buffer and incubated with a working solution consisting of fluorescein-labeled nucleotide mix and terminal deoxynucleotidyltransferase for 1 h at 37 °C in a humidified dark chamber. The reaction was ended by adding 2 × SSC solution. Nuclei were stained with Hoechst for 5 min. The coverslips were mounted with 75% glycerol/PBS and observed under a fluorescence microscope (Ti-E, Nikon). Release of lactate dehydrogenase (LDH) in culture supernatant was determined with a kit (Beyotime, Shanghai, China). The activity of LDH was estimated by measuring absorbance at 490 nm using a microplate reader.

### Reverse transcription-quantitative polymerase chain reaction (qRT-PCR)

Total RNA was isolated from cells and EVs by an RNAiso Plus kit (Takara, Dalian, China) following the supplier’s protocol. RNA pellets were resuspended in RNase-free H_2_O and quantified. RNA (500 ng) was reversely transcribed into cDNA using Mir-X miRNA qRT-PCR SYBR kit or PrimeScriptTMRT Master Mix (Takara) for miRNA or mRNA, respectively. Real-time PCR was performed using SYBR Premix Ex Taq II (Takara) and Quantstudio 5 (Bio-rad, Hercules, CA). The internal reference in miRNAs and mRNAs detection were U6 and β-actin, respectively. Primers included has-miR-1246, 5′-AATGGATTTTTGGAGCAGGAA; has-miR-1290, 5′-GTGGATTTTTGGATCAGGGAA; has-miR-486-3p, 5′-GGGCAGCTCAGTACAGGAT; has-miR-486-5p, 5′-TGTACTGAGCTGCCCCGAG; has-miR-320b, 5′-AAGCTGGGTTGAGAGGGCAA; has-miR-144-3p, 5′-CCTACAGTATAGATGATGTACT; has-miR-142-3p, 5′-TGTAGTGTTTCCTACTTTATGGA; has-miR-150-5p, 5′-TCCCAACCCTTGTACCAGTG; has-miR-223-3p, 5′-TGTCAGTTTGTCAAATACCCCA; has-miR-423-5p, 5′-GGGCAGAGAGCGAGACTTT; Cav-1, 5′-TCTACAAGCCCAACAACAAGG and 5′-AGGAAGGAGAGAATGGCAAAG; Flot-1, 5′-CGGAGGCCGAGTGTTTGTC and 5′-GTGGCGGGTATAAACCTTTTCA; Pak-1, 5′-GAAACACCAGCACTATGATTGGA and 5″-GAAACACCAGCACTATGATTGGA; Rac-1, 5′-GAGACGGAGCTGTTGGTAAAA and 5′-ATAGGCCCAGATTCACTGGTT; Dyn-2, 5′-TTTGGCGTTCGAGGCCATT and 5′-CAGGTCCACGCATTTCAGAC. The levels of miRNA and mRNA were normalized with U6 (5′-GGAACGATACAGAGAAGATTAGC and 5′-TGGAACGCTTCACGAATTTGCG) and β-actin (5′-CATCCGTAAAGACCTCTATGCCAAC and 5′-ATGGAGCCACCGATCCACA), respectively.

### Western blotting

Cells and EVs were lysed with working solution of the RIPA buffer (Beyotime) containing a protease inhibitor cocktail (Roche). Proteins were separated by sodium dodecyl sulfate–12% polyacrylamide gel (SDS–PAGE) electrophoresis and blotted onto polyvinylidene fluoride (PVDF) membranes (Millipore). Membranes were incubated with PBST (PBS and 0.1% Tween 20) containing 5% skim milk for 1 h, and successively incubated with primary antibodies overnight at 4 °C and secondary antibodies for 1 h at room temperature. Membranes were developed using an enhanced chemoluminescence (ECL) system (Clinx Science Instruments, Shanghai, China). The primary and secondary antibodies included β-actin (1:5000, Genetex), CD63, CD9, Alix, voltage-dependent anion-selective channel (VDAC) 1^37^, Calreticulin, Lamin A/C, Tau, Map-2 (1:1000, Abcam, Cambridge, UK), Cleaved caspase-3 (aCasp3), Cav-1 (1:1000, CST, Boston, MA), HRP-conjugated goat anti-rabbit IgG (Genshare, Xian, China) and HRP-conjugated goat anti-mouse IgG (HRP) (Genshare).

### Statistical analysis

All the statistical analyses were performed with Image Pro Plus 6.0, SPSS 16.0, and Graph Pad Prism 7.0 software. The unpaired and two-tailed Student’s *t*-test was used to determine statistical significance of most experiments. Some data that did not comply with normal distribution were analyzed by the Mann–Whitney test. All experiments were repeated at least for three times, and the number of repetition was indicated in the legends of each graph. Data were shown as mean ± SEM. *P* < 0.05 was considered as significant.

## Results

### HUVECs-derived exosomes protected neurons from oxygen-glucose depletion (OGD) injury

**Fig. 1:**
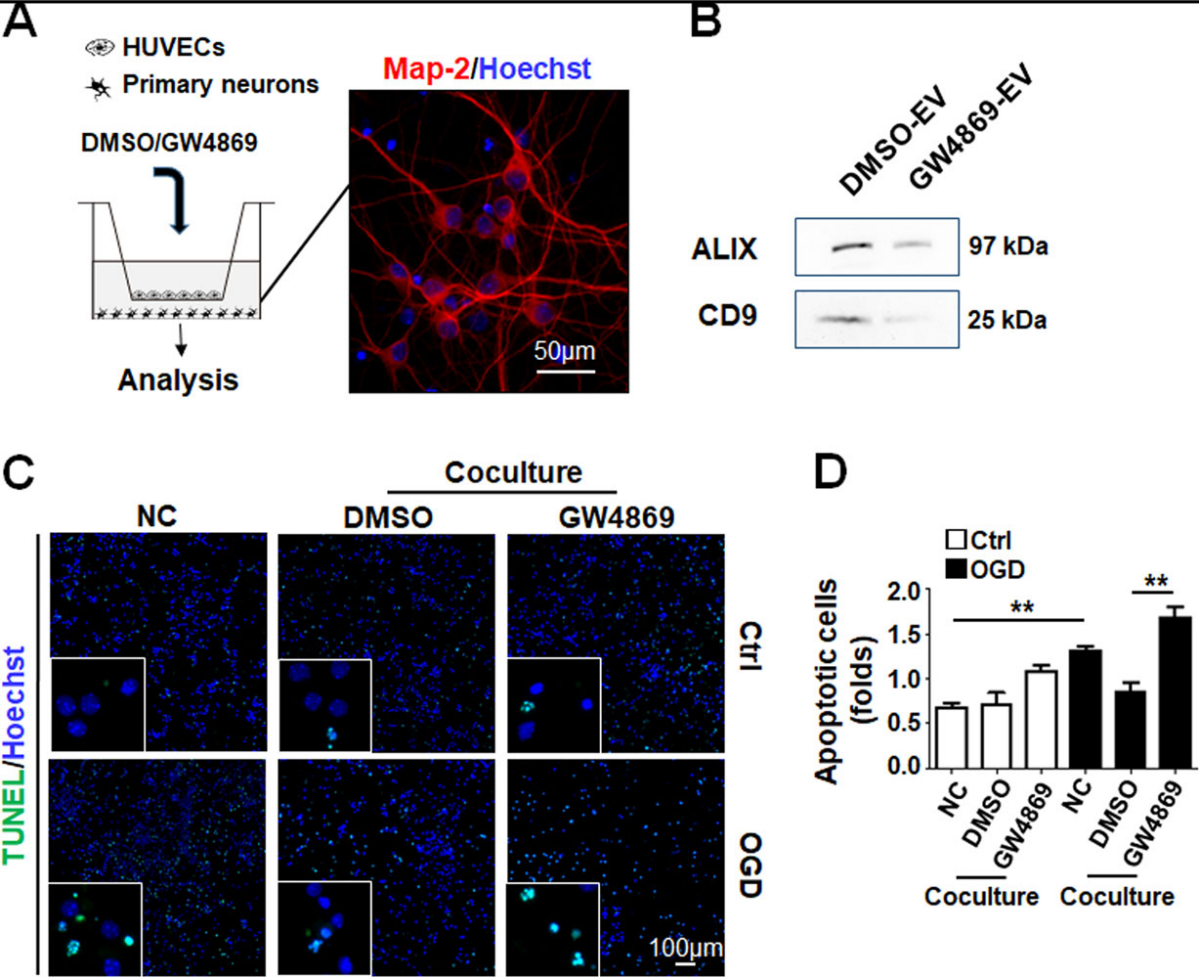
HUVECs-derived sEVs protected neurons from OGD injury in culture. **a** The coculture of HUVECs and neurons in a transwell system. Primary neurons were stained with MAP-2 and counter-stained with Hoechst. **b** EVs were precipitated from equal amounts of supernatants of the coculture and evaluated by Western blotting of the EV markers. **c**, **d** TUNEL staining of neurons in the coculture assay in the presence of DMSO or GW4869. Apoptotic neurons were shown (folds compared with the control) **d**. Bars = means ± s.e.m., *n* = 5. **P < 0.01.

To test the role of EVs in EC-mediated neuronal protection, we set up a coculture system of HUVECs and primary hippocampal neurons treated by OGD in the absence or presence of GW4869, a neutral sphingomyelinase inhibitor that blocks the secretion of exosomes^35^ (Fig. 1a). The blocking efficiency of GW4869 was confirmed by isolation of EVs followed by Western blotting (Fig. 1b). The result showed that OGD led to an increase of neuronal apoptosis, which was ameliorated by coculture with HUVECs. However, the protection effect of HUVECs was reduced by the addition of GW4869 (Fig. 1c, d), suggesting that HUVECs-mediated neuronal protection was likely via the secretion of exosomes, a kind of EVs.

**Fig. 2:**
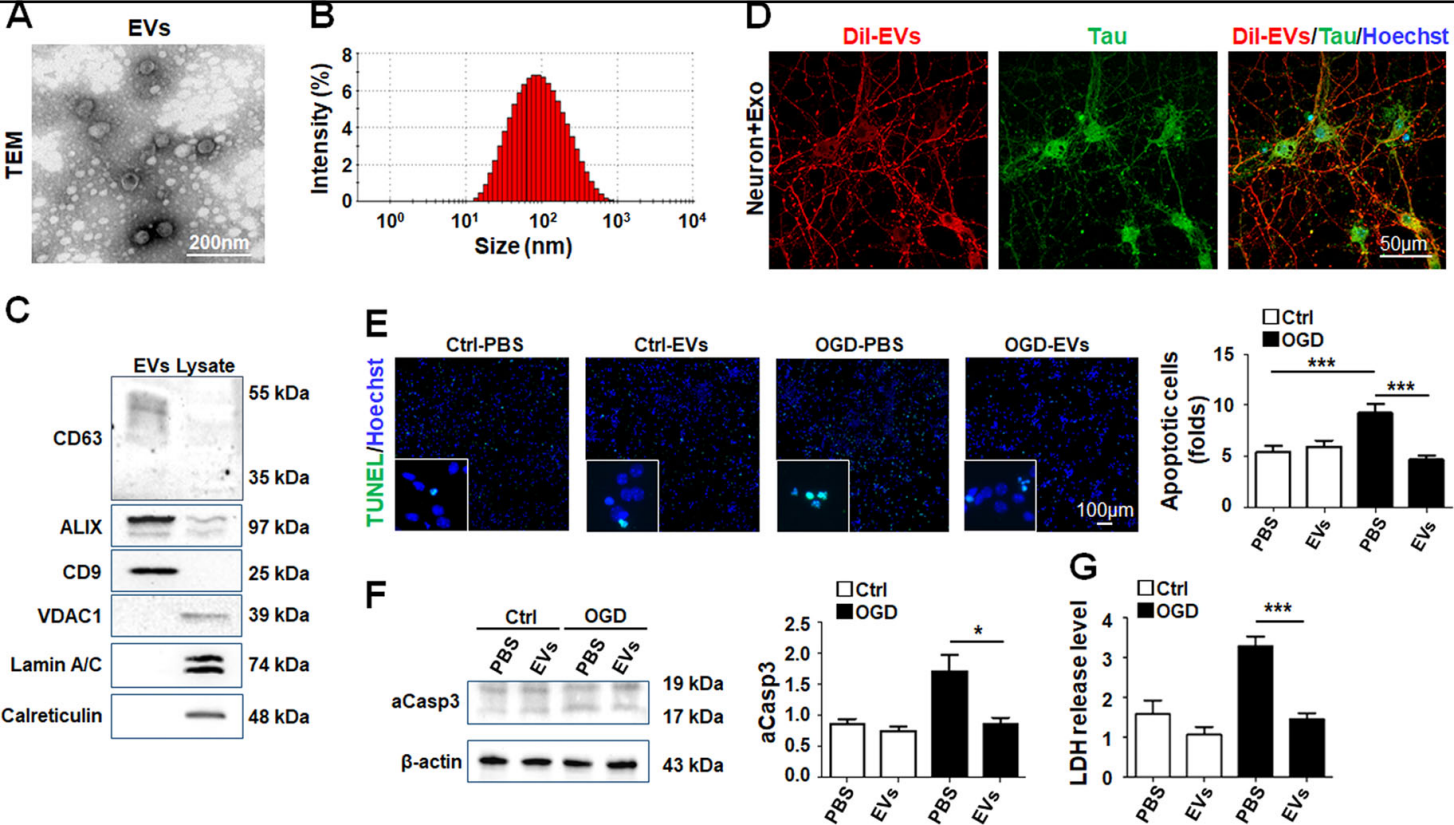
HUVECs-derived EVs showed a protective effect on OGD-treated neurons in a coculture assay. **a**–**c** Characterization of EVs isolated from HUVECs by transmission electronic microscope (TEM) **a**. The size of EVs was assessed by nano series-nano-ZS analysis **b**. In **c**, total proteins of EVs and HUVEC lysates were prepared, and the levels of CD63, CD9, Alix, VDAC1, Lamin A/C, and calreticulin were determined by Western blotting. **d** HUVECs-derived EVs were labeled with DiI and incubated with primary neurons for 12 h. Tau was stained to label axons in neurons. **e**–**g** Primary neurons were treated by OGD and incubated with PBS or EVs. Apoptosis was assessed by TUNEL assay **e**. The protein level of activated Caspase-3 (aCasp3) was determined by Western blotting **f** (*n* = 3). LDH release in the culture supernatant was evaluated **g**. Bars = means ± s.e.m, *n* = 5. *P < 0.05, ***P < 0.001.

To further investigate the effects of HUVECs-derived EVs on neurons, we isolated EVs from culture supernatant of HUVECs. HUVECs-derived EVs were vesicles of around 100 nm in size with a bilayer structure (Fig. 2a, b). Western blotting showed that EV markers including CD63, CD9, and ALIX were enriched in the EV preparations, but the negative markers VDAC1, calreticulin and lamin A/C were hardly detectable (Fig. 2c). We then incubated primary hippocampal neurons with HUVECs-derived EVs for 12 h. Neurons could efficiently intake DiI-labeled EVs in both cell bodies and neuronal processes (Fig. 2d). Then, primary hippocampal neurons were treated with OGD and incubated with HUVECs-derived EVs for 12 h. TUNEL staining showed that incubation with EVs could reduce neuronal apoptosis after OGD (Fig. 2e). Moreover, the levels of intracellular-activated caspase 3 and LDH release also decreased in OGD-treated neurons incubated with EVs (Fig. 2f, g). These results indicated that EVs from ECs attenuated neuronal apoptosis induced by OGD.

### miR-1290 carried by HUVECs-derived EVs protected OGD-insulted neurons

**Fig. 3:**
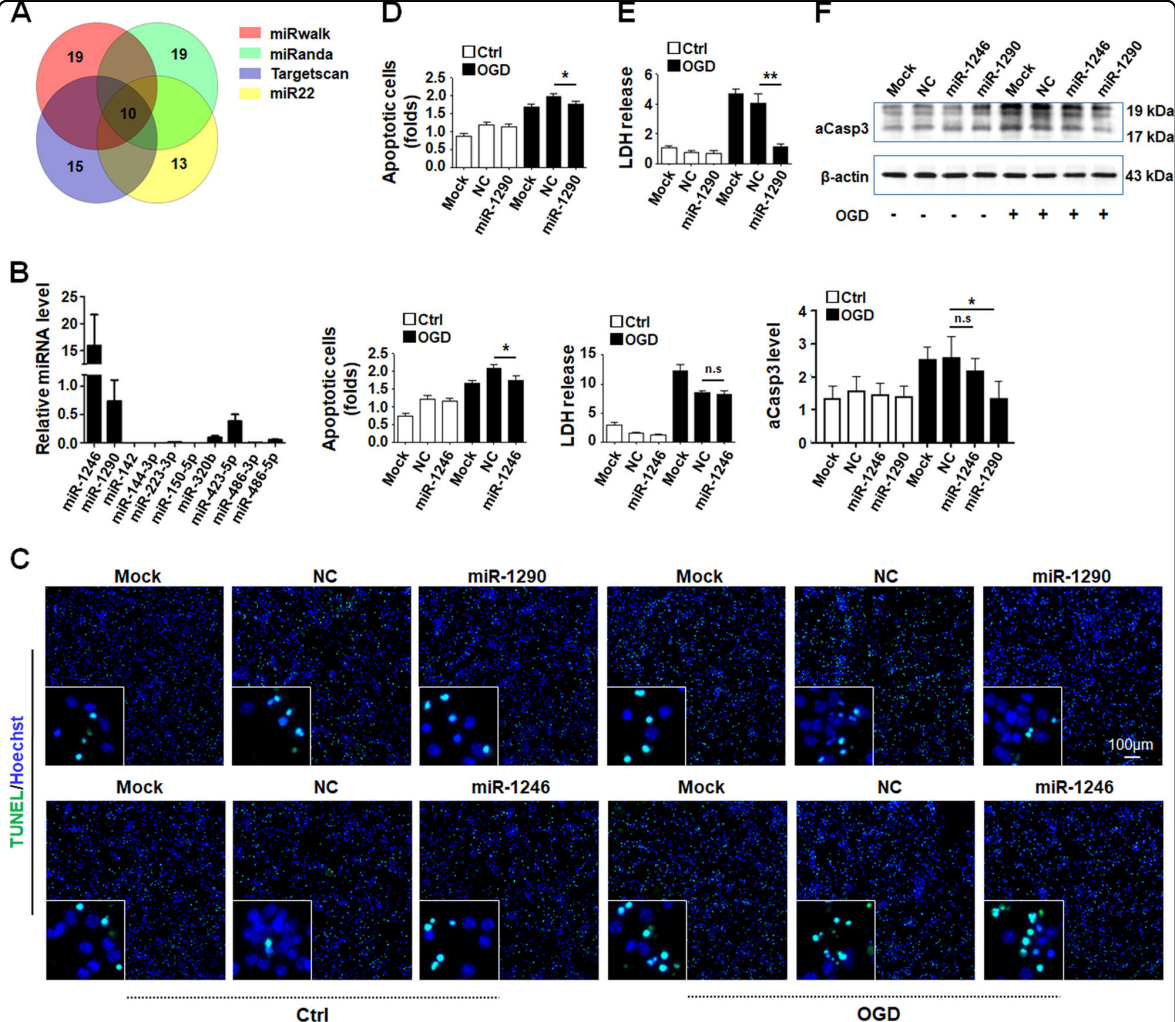
miR-1290 and miR-1246 in HUVECs-derived EVs exhibited protective effect on OGD-treated neurons. **a** EC-derived and apoptosis-related miRNAs in literatures were screened and illustrated with a Venn plot. **b** The levels of 10 miRNAs in **a** were determined in EVs derived from HUVECs. **c**, **d** Primary neurons were treated by OGD and incubated with control oligos (NC) or mimics of miR-1246 and miR-1290. The apoptosis of neurons was evaluated by TUNEL. **e** Cell death was evaluated by LDH release. **f** The protein level of aCasp3 in neurons from different groups was measured by Western blotting. Bars = means ± s.e.m, *n* = 3. *P < 0.05, **P < 0.01, n.s., not significant.

EVs exert their biological functions through delivering cargos they carry. To discover the mechanism of the anti-apoptosis role of HUVECs-derived EVs, we performed data-mining of sequencing data deposited in miRwalk, miRanda, Targetscan, and miR22 database for ECs-derived EVs miRNAs related with apoptosis. () Ten miRNAs were picked up as candidates based on these standards, and qRT-PCR showed that hsa-miR-1246 and hsa-miR-1290 were highly enriched in HUVECs-derived EVs (Fig. 3a, b). We then transfected primary neurons with miRNA mimics of hsa-miR-1246 and hsa-miR-1290 and determined apoptosis by TUNEL. Quantitative analysis showed that overexpression of miR-1246 or miR-1290 attenuated apoptosis in neurons suffering from OGD (Fig. 3c, d). However, examining LDH release and aCsp-3 level using LDH activity assay and Western blotting, respectively, showed that miR-1290 significantly reduced death of OGD-treated neurons, but miR-1246 had marginal activity without statistical significance (Fig. 3e, f). These data suggested that HUVECs-derived EVs protected OGD-treated neurons most likely through miR-1290, although the effect of miR-1246 could not be completely excluded.

### OGD treatment enhanced EV intake by neurons

**Fig. 4:**
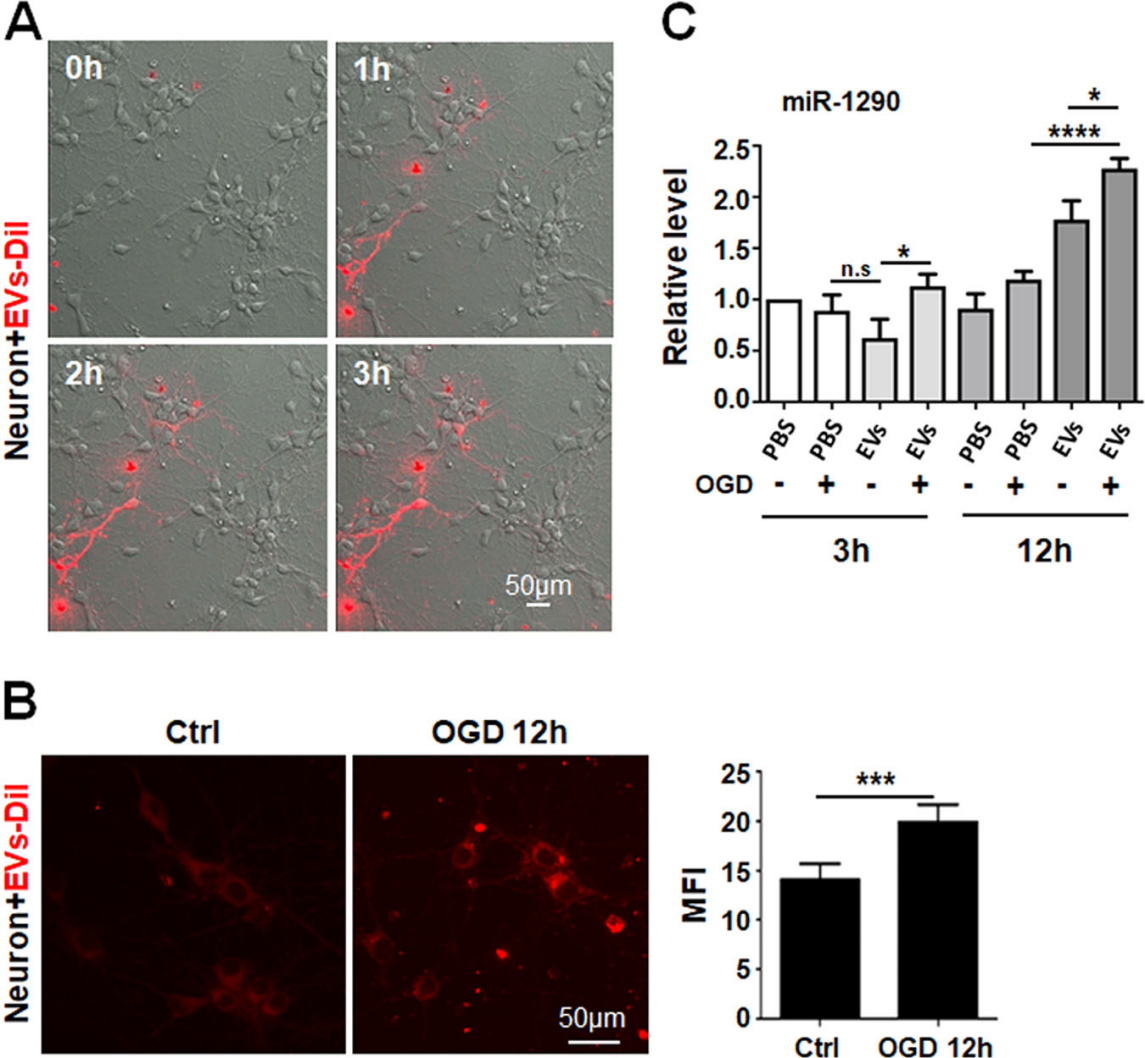
OGD-treated neurons displayed enhanced internalization of EVs derived from HUVECs. **a** HUVECs-derived EVs were labeled with DiI, and incubated with primary neurons. The internalization of DiI-EVs was recorded by live imaging, and shown at 0, 1, 2, and 3 h. **b** DiI-EVs were incubated with neurons from control group or OGD group for 12 h and observed under a fluorescence microscope. The mean fluorescence intensity (MFI) was determined to compare the difference of fluorescence intensity between the two groups (*n* = 3). **c** The level of miR-1290 in neurons incubated with HUVECs-derived EVs for 3 or 12 h was determined (*n* = 6). Bars = means ± s.e.m. *P < 0.05, ***P < 0.001.

Next, to evaluate the dynamics of EV intake by neurons, we labeled HUVECs-derived EVs with DiI, and incubated primary neurons with DiI-labeled EVs for different periods of time. Live cell imaging showed that neurons could take in a significant amount of DiI-EVs as early as 1 h after incubation, and EV intake appeared reaching its maximum at about 3 h after incubation (Fig. 4a). Interestingly, quantification of intracellular fluorescence signal showed that OGD treatment enhanced intake of DiI-labeled EVs by neurons (Fig. 4b). Consistently, the level of miR-1290 was significantly higher in neurons incubated with HUVECs-derived EVs after OGD-treatment (Fig. 4c). A similar tendency was observed in miR-1246 (Supplementary Fig. S2). These data suggested that OGD-treated neurons could enhance intake of EVs, which likely serves as a mechanism to increase their survival under stress environment.

### Cav-1 was significantly upregulated in neurons after OGD in vitro and in vivo

**Fig. 5:**
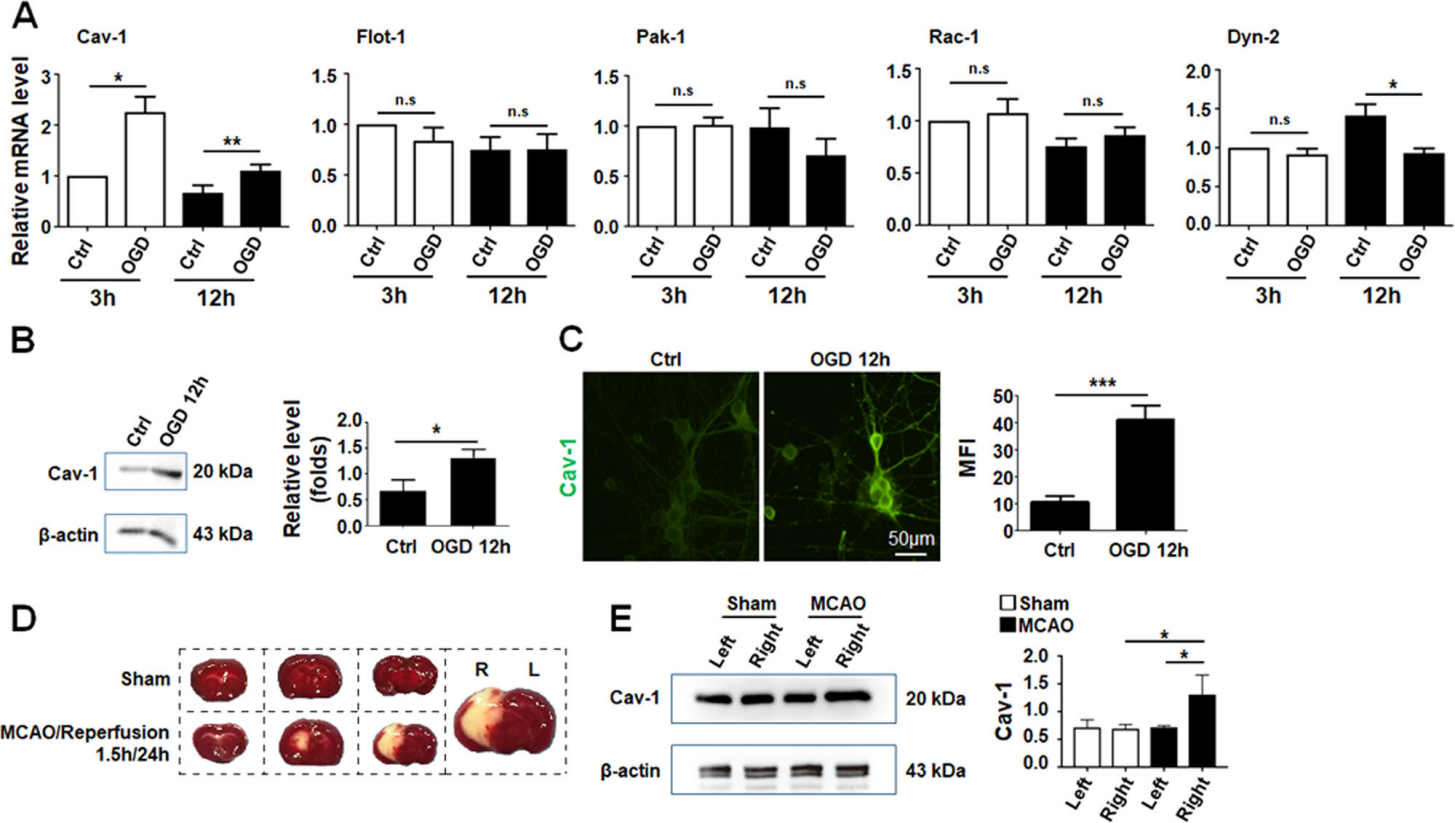
Cav-1 was significantly upregulated in neurons upon OGD treatment. **a** Primary neurons were treated with OGD for 1.5 h and incubated with HUVECs-derived EVs for 3 or 12 h. The mRNA levels of Cav-1, Flot-1, Pak-1, Rac-1, and Dyn-2 were determined using qRT-PCR. **b**, **c** Protein level of Cav-1 in neurons at 12 h in **a** was determined by Western blotting **b** and immunofluorescence **c**. **d**, **e** Mice were treated with MCAO-reperfusion. TTC staining was performed to show the ischemic regions (pale) **d**. Tissue lysates were prepared, and the protein level of Cav-1 in the damaged hemisphere and control side was determined using Western blotting **e**. Bars = means ± s.e.m., *n* = 3. *P < 0.05, **P < 0.01, ***P < 0.001, n.s. not significant.

We then tried to further assess the molecular mechanism for increased EV intake by neurons after OGD. Several molecules of the endocytosis pathway including Flot-1, Cav-1, Pak-1, Rac-1, and Dyn-2 have been implicated in exosomal intake in various types of cells^14,38^. Total RNA was prepared from primary neurons suffered from OGD. qRT-PCR showed that OGD treatment significantly upregulated Cav-1 expression at both mRNA and protein levels (Fig. 5a, b). Immunofluorescence confirmed that OGD treatment induced the expression of Cav-1 in neurons (Fig. 5c). The expression of other exosomal intake-related molecules including Flot-1, Pak-1, Rac-1, and Dyn-2 was not influenced by OGD as determined by qRT-PCR (Fig. 5a). Furthermore, we established MCAO-reperfusion model in mice (Fig. 5d). Western blotting indicated that Cav-1 was predominantly upregulated in the injured cerebral hemisphere (Fig. 5e). These findings suggested that Cav-1 was upregulated after IRI in neurons, which could be responsible for increased EV intake by neurons.

### Cav-1-mediated EV intake through Cav-1-dependent endocytosis pathway

**Fig. 6:**
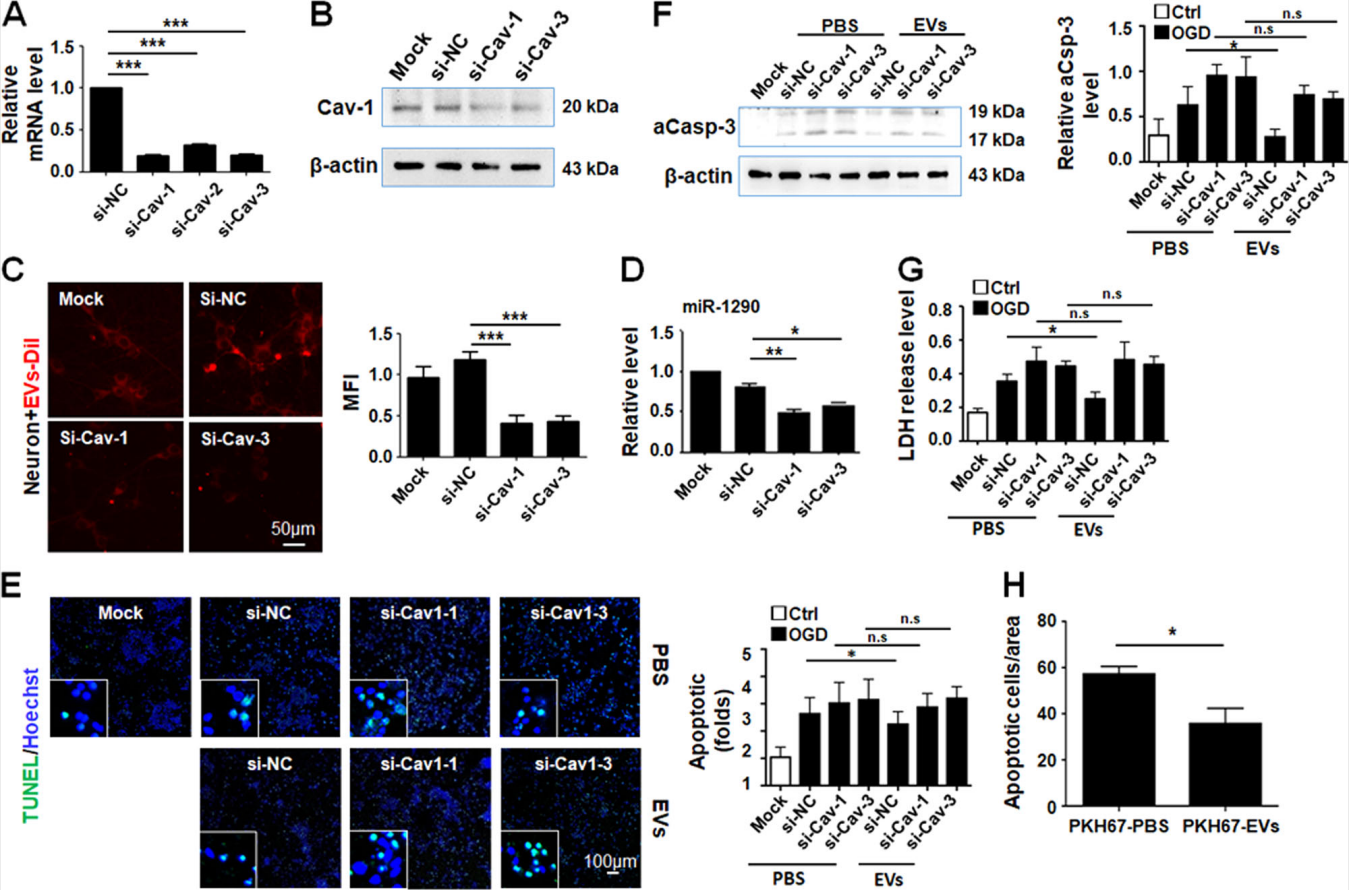
Cav-1 was responsible for increased internalization of HUVECs-derived EVs by neurons treated by OGD. **a** Neurons were transfected with three different siRNAs targeting Cav-1. The mRNA level of Cav-1 was determined by qRT-PCR. **b** The efficiency of si-Cav-1 and si-Cav-3 was further confirmed by Western blotting. **c**, **d** Primary neurons were transfected with si-NC, si-Cav-1, or si-Cav-3, and treated with OGD and incubated with HUVECs-derived EVs for 12 h. Cells were observed under a fluorescence microscope, and the mean fluorescence intensity of DiI-labeled EVs in neurons was measured **c**. miR-1290 was determined in different groups **d**. **e**–**g** Knockdown of Cav-1 abrogated EVs-mediated neuronal protection in OGD, as determined by aCasp-3 Western blotting **e**, TUNEL **f**, and LDH release **g**. **h** Male mice of 6–8 weeks old were injured by MCAO. Immediately after the MCAO operation, mice were injected intracranially with 1 μL of pKH67-PBS or pKH67-sEVs at the site adjacent to the hippocampus of the impaired hemisphere under the navigation of a murine brain stereotaxic apparatus. The brain samples were sectioned and stained routinely, photographed under a fluorescence microscope, and apoptotic cells of CA1–CA2 were quantitatively compared according to Supplementary Fig. S1. The injection regions that were too far or too close to the hippocampus were excluded from analysis (*n* = 5 for PBS and *n* = 6 for EVs). Bars = means ± s.e.m., *n* = 3. *P < 0.05, **P < 0.01, ***P < 0.001, n.s., not significant.

To determine whether Cav-1 was involved in EV intake in OGD-treated neurons, siRNAs to Cav-1 were synthesized, which could suppress Cav-1 expression at both mRNA and protein levels in neurons after transfection (Fig. 6a, b). Then, primary neurons transfected with Cav-1 siRNA or control were treated with OGD and incubated with DiI-labeled EVs derived from HUVECs. We found that transfection of Cav-1 siRNA resulted in obvious reduction of DiI-EVs in neurons as determined by MFI (Fig. 6c). The level of EV-enriched miR-1290 was also significantly lower in neurons transfected with si-Cav-1 compared with those transfected with the control siRNA (Fig. 6d). Moreover, OGD-induced apoptosis, which was attenuated by incubation with HUVECs-derived EVs, was restored in the presence of Cav-1 siRNAs (Fig. 6e–g). These results suggested that neurons under IRI could increase their survival by upregulating Cav-1 to enhance intake of EVs derived from ECs. Lastly, we injected HUVEC-derived EVs directly into brain tissues of mice injured by MCAO. TUNEL staining showed that HUVEC-derived EVs could attenuate apoptosis in MCAO-injured brain tissues (Fig. 6h; Supplementary Fig. S1), suggesting that EC-derived EVs could serve as a promising therapy for IRI in brain.

## Discussion

EVs have exhibited promising therapeutic potentials in the treatment of stroke, and a panel of clinical trials have been reported^18,32,39–41^. Moreover, studies have also indicated that functionally modified EVs could be utilized to deliver drugs and specifically target neural cell^18,42^. So far, EVs derived from various cell types such as MSCs have been suggested to potentially benefit patients by different mechanisms including reduction of edema, immunomodulation, and promoting neurogenesis, and angiogenesis^37,38,43–45^. ECs regulate tissue homeostasis and participate in tissue injury and regeneration through angiocrine. It has been shown that BDNF, NGF, and VEGF secreted by ECs could promote neuronal survival and angiogenesis^24,28,46^. ECs-derived EVs, which represent a novel type of angiocrine, have been shown to be protective in various human diseases such as cardiovascular diseases^47^. Atherosclerotic conditions could promote the package of miR-92a-3p into endothelial microvesicles and thereby regulates angiogenesis^48^. We have recently demonstrated that ECs-derived EVs protect NSCs from apoptosis and promotes stemness^31^. In this study, we further extend our observation to primary hippocampal neurons under OGD, which mimics IRI in stroke. Our data showed that cultured neurons could efficiently internalize EVs, and ECs-derived EVs attenuate apoptosis in neurons under OGD. Therefore, ECs-derived EVs could play protective roles in different stages of stroke, namely protect neurons from cell death and promote regeneration by increasing survival and activity of NSCs. Considering that ECs could be relatively easily cultured ex vivo, these properties of ECs-derived EVs make them attractive candidates as a tool for therapy of stroke. Interestingly, internalized EVs are localized at both cell body and processes of neurons. EVs have been proved to be transported from a neuron to the next one through axon by hijacking the endosomal system^49^. In our condition, whether this characteristic intracellular distribution pattern reflects different effects of EVs on neurons requires further investigation.

Although different components of EVs including proteins, RNAs, and lipids have been implicated in their functions, a large amount of studies have pointed to that miRNAs may play a critical role in transmitting signals by EVs between cells under many different experimental settings^38,39,47,50–52^. Xin et al. showed that miRNA miR-17-92 cluster in exosomes enhance neuroplasticity and functional recovery after stroke in rats. It has also been shown that MVP-mediated exosomal sorting of miR-193a promotes colon cancer progression^50,52^. ECs-derived EVs contain many different types of miRNAs^8^. By bio-informatic mining of published data, we identified two apoptosis-related miRNAs, miR-1246 and miR-1290, were enriched in EC-derived EVs. Further molecular characterization has shown that miR-1290 can attenuate apoptosis of primary neurons treated by OGD, although miR-1246 also exhibited similar effect but at a weaker strength as compared with miR-1290. Both miR-1290 and miR-1246 have been implicated in regulating cancer cell proliferation and migration, and exosomal miR-1290 and miR-1246 might serve as tumor biomarkers^53–57^. Our data have shown that these two miRNAs are highly enriched in ECs-derived EVs, supporting a neuronal protective role of ECs-derived EVs in stroke. However, our data do not completely exclude the contribution of other EV components in EV-mediated neuronal protection. The contribution of components, such as lipids, proteins, and mRNAs, in EVs to neuronal survival is worth of further investigation.

It is interesting that OGD treatment increased exosomal intake by neurons. This finding suggests that neurons under stress, such as IRI in this study, could actively confront the insults or release stress by increasing intake of EVs from their microenvironment. EVs enter recipient cells through direct fusion, receptor-dependent endocytosis and phagocytosis^9^. In our study, we identified Cav-1 as a neuronal receptor for intake of ECs-derived EVs, and OGD treatment upregulates Cav-1 expression in neurons. Knockdown of Cav-1 could reduce neuronal intake of EVs and overwhelm EVs-mediated neuronal protection in OGD. It has been reported that the interference of endocytosis-related molecules, such as Cav-1 and Dyn-2 could disturb the internalization of EVs^14,38^. Moreover, Cav-1 has been shown to be protective in stroke. Cav-1 deficiency increases cerebral ischemic injury, and regulation of Cav-1 expression determines early brain edema after experimental focal cerebral ischemia^58,59^. Choi et al. reported that overexpression of Cav-1 attenuates brain edema by inhibiting tight junction degradation^59^. In addition, Cav-1 may also participate in neural protection via N-methyl-Daspartate receptor-mediated Src and extracellular signal-regulated kinase 1/2 activation^60^. Our data put forward a novel mechanism of Cav-1-mediated neuronal protection, namely Cav-1 play its neuronal protective role through mediating exosomal intake in ischemic injury. These findings provide further support for therapeutic use of EVs in the treatment of stroke.

## Supplementary information

Supplementary Figure Legends

Figure S1

Figure S2

Declaration of contributions TO ARTICLE
