# Supplementary material for: Neurons can upregulate Cav-1 to increase intake of endothelial cells-derived extracellular vesicles that attenuate apoptosis via miR-1290
Source: Cell Death Dis. 2019 Nov 18;10(12):869. doi: 10.1038/s41419-019-2100-5 (PMC6861259; doi:10.1038/s41419-019-2100-5)
Supplement: Supplementary file 1 — Supplementary Figure Legends [file 41419_2019_2100_MOESM1_ESM.docx]

**Supplementary Figure Legends**

**Fig. S1. Quantification of apoptotic cells in CA1-CA2 of hippocampus after MCAO and intracranial injection of sEVs.** Male mice of 6-8 weeks-old were injured by MCAO. Immediately after the MCAO operation, mice were injected with 1 μl of pKH67-PBS or pKH67-sEVs at the site adjacent to the hippocampus of the impaired hemisphere under the navigation of a murine intracranial injection system. The brain samples were sectioned and stained routinely and photographed under a fluorescence microscope. The injection sites (in green) were marked by a circle with white dashed line, and samples with correct injection sites were analyzed further. With the injection site as the center, a large circle with yellow dashed line was made to include the CA1-CA2 of hippocampus. Apoptotic cells of CA1-CA2 within the yellow circle were counted and compared.

**Fig. S2. OGD treatment could enhance the internalization of miR-1246, another abundant miRNA in HUVEC-EVs, in neurons.** HUVECs-derived EVs were incubated with primary neurons for 3 or 12 h. The level of miR-1246 in neurons was determined by qRT-PCR (n = 6). Bars = means ± s.e.m. **, P < 0.01, ****, P < 0.0001.
