# Supplementary figures and images for: Neurons can upregulate Cav-1 to increase intake of endothelial cells-derived extracellular vesicles that attenuate apoptosis via miR-1290
Source: Cell Death Dis. 2019 Nov 18;10(12):869. doi: 10.1038/s41419-019-2100-5 (PMC6861259; doi:10.1038/s41419-019-2100-5)

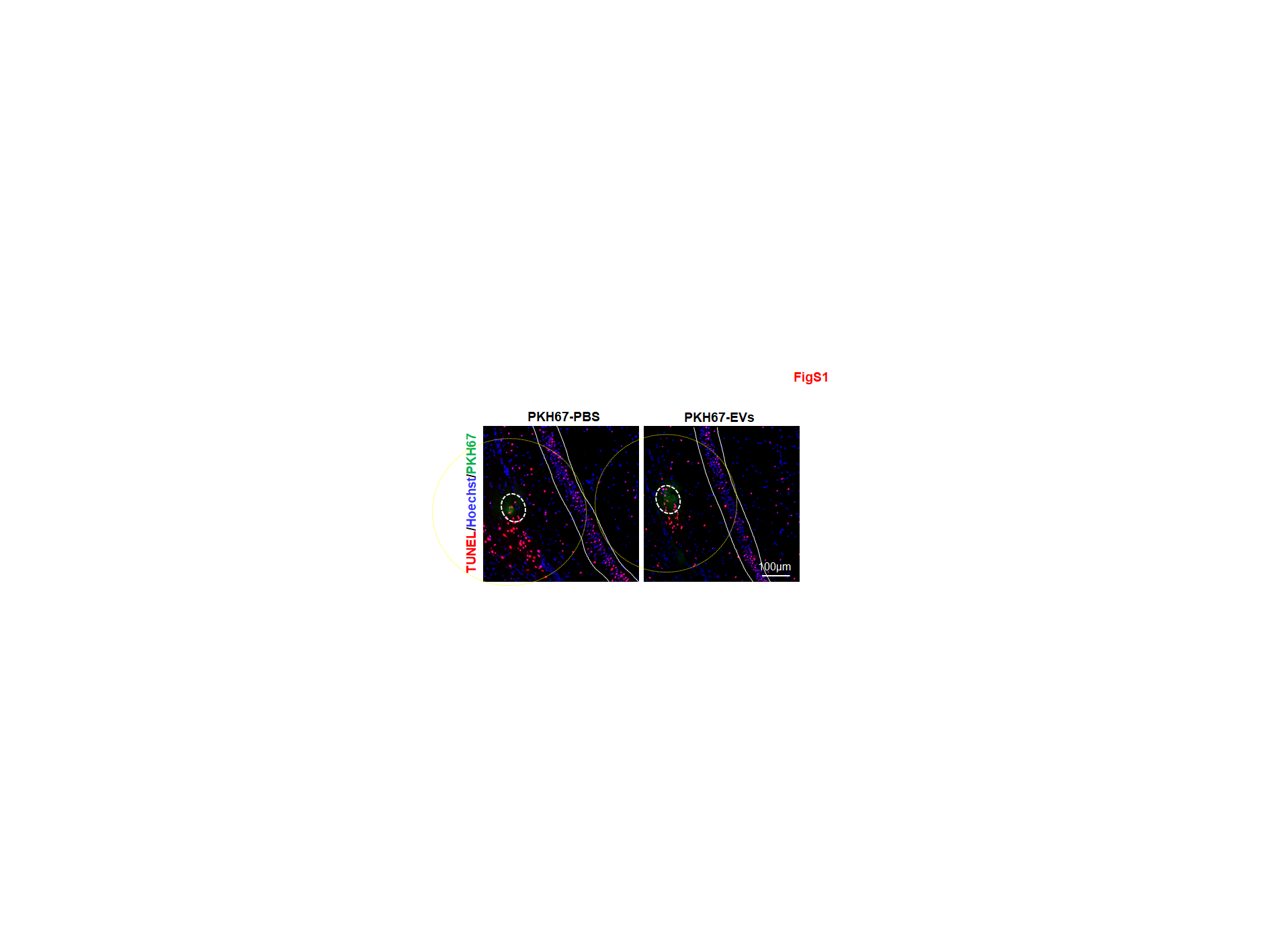

Supplement: Supplementary file 2 — Figure S1 [file 41419_2019_2100_MOESM2_ESM.tif]

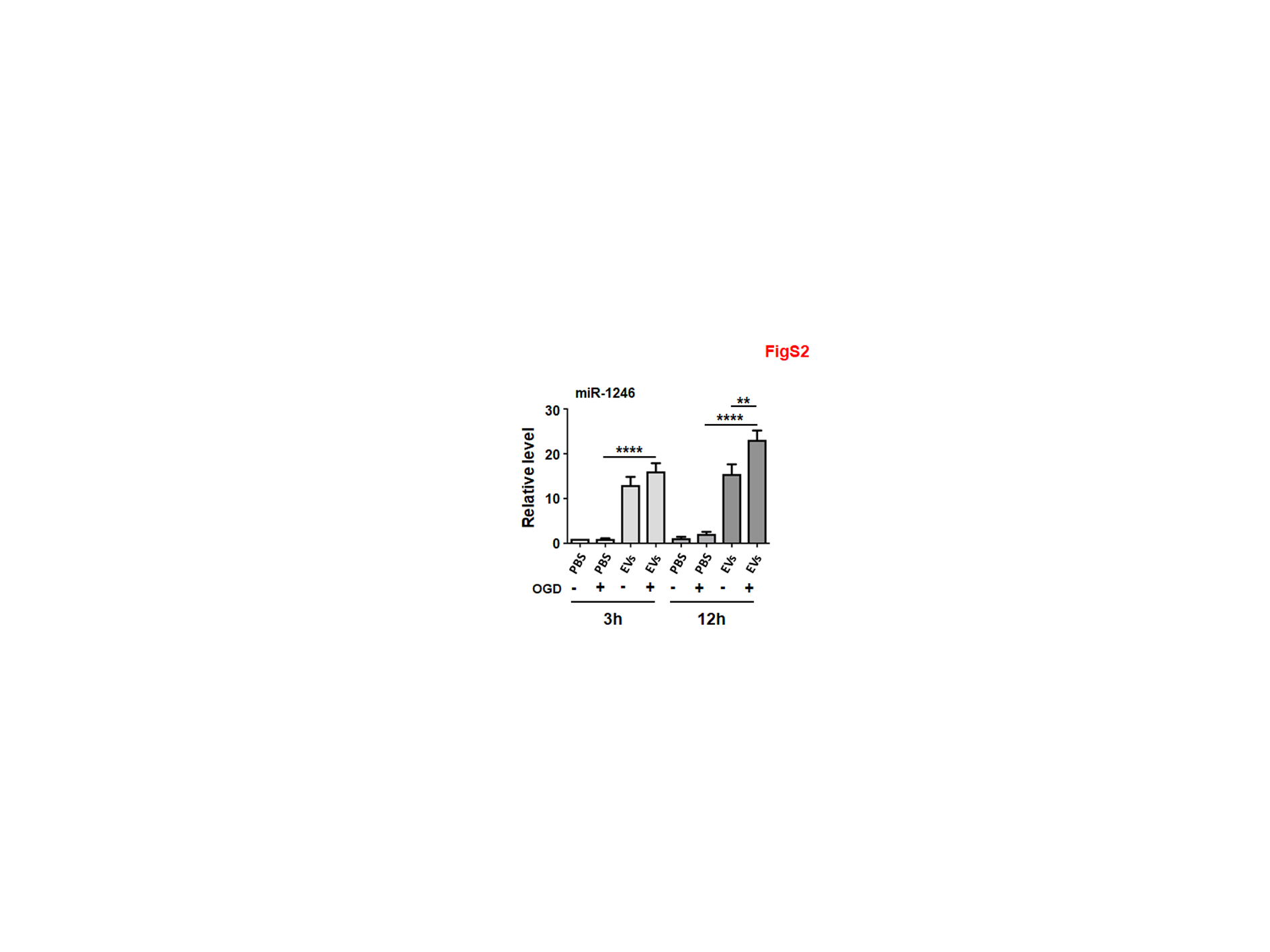

Supplement: Supplementary file 3 — Figure S2 [file 41419_2019_2100_MOESM3_ESM.tif]
