# Supplementary material for: Neurons can upregulate Cav-1 to increase intake of endothelial cells-derived extracellular vesicles that attenuate apoptosis via miR-1290
Source: Cell Death Dis. 2019 Nov 18;10(12):869. doi: 10.1038/s41419-019-2100-5 (PMC6861259; doi:10.1038/s41419-019-2100-5)
Supplement: Supplementary file 4 — Declaration of contributions TO ARTICLE [file 41419_2019_2100_MOESM4_ESM.pdf]

DECLARATION OF CONTRIBUTIONS TO ARTICLE

ADMC

Manuscript Number:	Journal Name:	
CDDIS-19-1666R	*Cell Death & Differentiation*	(the 'Journal')

Proposed Title of the Contribution:

Contributions (the 'Contribution)

Author(s):

Kang-Yi Yue, Pei-Ran Zhang, Min-Hua Zheng, Xiu-Li Cao, Yuan Cao, Yi-Zhe [illegible] (the 'Authors')

For all *CDD* articles, each person named as an author in the published version must be able to show he or she has contributed substantially to the article.

Authorship credit should be based on 1) substantial contributions to conception and design, acquisition of data, or analysis and interpretation of data; 2) drafting the article or revising it critically for important intellectual content; and 3) final approval of the version to be published. Authors should meet conditions 1, 2 and 3.

Any person who cannot be shown to have made a substantial contribution to the article cannot be listed as an author in the final version. The name of any person who is deemed to have made a minor contribution can, however, appear in the Acknowledgments section of the article.

Please complete the table below to indicate the contributions of all named authors to the manuscript.

Author Full Name:	Specification of Contribution to the Manuscript:
Kang-Yi Yue	Perform MCAO on mice and most of the in vitro experiments
Pei-Ran Zhang	Perform stereotactic injection in the MCAO mice
Min-Hua Zheng	Maintain mice, and perform immunofluorescence staining
Xiu-Li Cao	Culture primary neurons and conduct statistical analysis
Yuan Cao	Help make frozen sections of hippocampus
Yi-Zhe Zhang	Help isolate the extracellular vesicles
Yu-Fei Zhang	Provide technical assistance for live cell imaging
Hai-Ning Wu	Acquire HUVECs from human umbilical vein
Zhi-Hong Lu	Acquire reagents and fund
Liang Liang	Write the original draft and supervise
Xiao-Fan Jiang	Design the experiments, and acquire fund
Hua Han	Design the experiment ,and edit the paper

Please complete the table below to indicate the contributions of all named authors to the figures.

Figure 1:

Figure 1A-1C: Kang-Yi Yue, Xiu-Li Cao
Figure 1D: Yuan Cao

Figure 2:

Figure 2A,B,C,E,F,G: Kang-Yi Yue, Xiu-Li Cao
Figure 2D: Min-Hua Zheng

Figure 3:

Figure 3A-F: Kang-Yi Yue, Xiu-Li Cao

Figure 4:

Figure 4A: Yu-Fei Zhang
Figure 4B,C: Kang-Yi Yue

Figure 5:

Figure 5A: Kang-Yi Yue
Figure 5B-E: Kang-Yi Yue, Min-Hua Zheng

Figure 6:

Figure 6A-G, S2: Kang-Yi Yue
Figure 6H,S1: Pei-Ran Zhang, Yuan Cao

Signed for and on behalf of the Author(s):	Print Name:	Date:
Liang Liang	Liang Liang	19/10/8
